# Customized osteosynthesis plates for multi-species fractures using ICP and TPS: an integrated CT imaging and computational modeling framework

**DOI:** 10.3389/fvets.2025.1700901

**Published:** 2025-10-30

**Authors:** Mohamed Amine Fares, Najah Elmounedi, Aicha Mouane, Ayomide Victor Atoki, Mohammed Messaoudi

**Affiliations:** ^1^Saharan Area Laboratory for Agricultural Modernization and Advancement (SALAMA - LAB), Higher School of Saharan Agriculture, El Oued, Algeria; ^2^Cell Therapy and Experimental Surgery of Musculoskeletal System LR18SP11 Lab, Faculty of Medicine, University of Sfax, Sfax, Tunisia; ^3^Department of Biology, Faculty of Nature and Life Sciences, University of El Oued, El Oued, Algeria; ^4^Department of Biochemistry, Kampala International University, Ishaka, Uganda; ^5^Laboratoire de Recherche sur les Produits Bioactifs et Valorisation de la Biomasse, Département de Chimie, ENS Kouba, Algiers, Algeria

**Keywords:** customized osteosynthesis plates, iterative closest point, thin-plate spline warping, 3D modeling, biomechanical stability

## Abstract

**Introduction:**

This study addresses the challenges of anatomical diversity and precision in orthopedic surgery by introducing a novel computational methodology for designing customized osteosynthesis plates. The goal is to improve anatomical fitting and surgical outcomes across different species.

**Methods:**

High-resolution computed tomography (CT) scans were used to generate 3D reconstructions of fractured bones, which were converted into point clouds. The Iterative Closest Point (ICP) algorithm was applied to minimize Euclidean distances between bone and plate models, ensuring optimal alignment. Subsequently, thin-plate spline (TPS) warping was employed to refine the adaptation of plates to complex bone geometries, enhancing biomechanical stability. The methodology was applied to bone scans from camels, dogs, and cats.

**Results:**

The customized plates achieved significantly improved anatomical fitting compared to conventional approaches, with reduced post-process distances and decreased operation times. The improved fitting was strongly correlated with enhanced surgical precision and stability.

**Discussion:**

The proposed workflow demonstrates high potential for improving fracture fixation in both human and veterinary medicine. By integrating CT imaging and computational modeling, this approach enhances efficiency, precision, and clinical outcomes in orthopedic surgery. Future work will refine the methodology and involve extensive clinical trials across species and fracture types.

## 1 Introduction

The advancement of imaging and computational technologies has significantly enhanced the field of orthopedic surgery, particularly in the customization of osteosynthesis plates for fracture fixation. Precise anatomical fitting is crucial for ensuring biomechanical stability and promoting optimal healing outcomes. This study leverages high-resolution computed tomography (CT) imaging and sophisticated computational workflows to develop customized osteosynthesis plates for various species, including mammals and avians. By utilizing state-of-the-art software tools and high-performance hardware, this research aims to improve the accuracy and effectiveness of fracture treatments (1).

High-resolution CT imaging provides detailed insights into bone morphology, which is essential for creating accurate three-dimensional (3D) reconstructions of fractured bones. This imaging technique has been extensively validated for its accuracy and reliability in clinical and research settings (2, 3). The data, stored in Digital Imaging and Communications in Medicine (DICOM) format, are converted into Standard Tessellation Language (STL) format using 3D Slicer (4), facilitating the segmentation and reconstruction of bone geometry (5). These models serve as the foundation for designing osteosynthesis plates that conform to specific anatomical requirements.

One of the major challenges in orthopedic surgery is the precise alignment and fitting of osteosynthesis plates to the bone surface. Standard plates often fail to conform perfectly to the intricate and variable geometry of bones, leading to suboptimal fixation, increased operation times, and potentially poor healing outcomes (6, 7). This issue is compounded in veterinary medicine, where the anatomical diversity among species necessitates highly customized solutions. The traditional manual methods for designing and fitting these plates are not only time-consuming but also prone to inaccuracies.

This study proposes an advanced computational methodology for designing customized osteosynthesis plates using Iterative Closest Point (ICP) and thin-plate spline (TPS) warping techniques, offering a solution to the challenges of achieving precise anatomical fitting in fracture fixation (8). The workflow involves several key stages to ensure optimal customization and biomechanical stability. Initially, high-resolution CT scans are utilized to generate 3D models of fractured bones, which are converted from DICOM to STL format using 3D Slicer, facilitating detailed bone segmentation. Next, mesh cleaning and point cloud generation are performed using tools like MeshLab and Python libraries such as numpy, open3d, and SciPy to refine the mesh and create dense point clouds essential for accurate alignment and surface adaptation (9, 10). The ICP algorithm is then employed to achieve precise alignment between the bone and plate models, minimizing the Euclidean distance between corresponding points, thereby ensuring an optimal fit (11). Following this, TPS warping is applied to further refine the plate’s fit to the bone’s complex curvature, ensuring minimal gaps and enhanced biomechanical performance. This method of surface adaptation leverages the flexibility of TPS to accommodate intricate bone geometries, ultimately improving the overall alignment and stability of the osteosynthesis plates. The integration of these advanced computational techniques aims to enhance the effectiveness of fracture treatments across various species, providing a robust framework for developing customized medical implants that conform to unique anatomical features. The proposed solution not only demonstrates the potential of interdisciplinary approaches in advancing healthcare technologies but also underscores the importance of precision in orthopedic surgery to improve patient outcomes. Through meticulous data acquisition, preprocessing, and computational modeling.

Recent studies have demonstrated the efficacy of patient-specific implants and 3D-printed medical devices in improving surgical outcomes (12, 13). However, while detailed morphometric studies continue to characterize the unique anatomy of specific veterinary species (14).

This research not only contributes to the field of orthopedic surgery by providing a comprehensive methodological framework but also demonstrates the potential of advanced computational techniques in achieving precise and reliable customization of medical implants. The findings underscore the importance of interdisciplinary approaches in advancing healthcare technologies and improving patient outcomes. The integration of high-resolution imaging, computational modeling, and additive manufacturing represents a significant step forward in the development of customized orthopedic solutions.

This study presents a novel approach to the design and validation of customized osteosynthesis plates, leveraging cutting-edge technologies to address the challenges of anatomical diversity and precision in orthopedic surgery. The proposed methodology has the potential to significantly improve surgical outcomes, reduce operation times, and enhance the overall quality of care in both human and veterinary medicine.

## 2 Materials and methods

### 2.1 Sample collection and imaging

#### 2.1.1 Sample collection

Bone scans were collected from various species including canines, felines, and camelines, some encompassing a femur bone fracture.

### 2.2 Imaging and data acquisition

High-resolution computed tomography (CT) scans and 3D models of bones were obtained from the femur bones of various species, including 10 camels, eight dogs, and three cats. The CT datasets were stored in the Digital Imaging and Communications in Medicine (DICOM) format. The DICOM files were then used for the three-dimensional (3D) reconstruction of the bone geometry, providing a detailed representation of the bone structure.

### 2.3 3D model preparation

#### 2.3.1 Osteosynthesis plates

Osteosynthesis plates, designed to stabilize fractures plates were modelized into Standard Tessellation Language (STL) format. The STL models either conformed to standard surgical dimensions or were customized to fit specific anatomical requirements of the species under study.

#### 2.3.2 Computational workflow

The computational workflow employed in this study was sophisticated, involving multiple specialized software tools and high-performance hardware to ensureprecision and efficiency. Initially, 3D Slicer (Version 5.0) (4) was used to convert DICOM files into STL format and to segment the bone structures from the CT data. This step was crucial for creating accurate 3D models of the fractured bones (15, 16).

Following segmentation, MeshLab (Version 2022.02) (9) was utilized for mesh cleaning, STL to XYZ conversion, and initial alignment of the bone models. MeshLab facilitated the refinement of the bone geometries, ensuring that the models were suitable for subsequent analysis.

For the processing of point clouds and the implementation of the iterative closest point (ICP) algorithm, a suite of Python libraries were employed. These included numpy (Version 1.22) (17), open3d (Version 0.16) (18), and SciPy (Version 1.8) (19). These libraries were instrumental in aligning and comparing the 3D models, providing the computational framework necessary for detailed analysis.

#### 2.3.3 Data acquisition and preprocessing

CT scan data in DICOM format were imported into 3D Slicer. Using threshold-based techniques, bone segmentation was performed to isolate the bone from surrounding tissues accurately. The segmented bone model was then exported as an STL file, facilitating further processing and analysis.

#### 2.3.4 Mesh cleaning

The bone STL file underwent processing in MeshLab to remove artifacts and ensure a smooth surface. This step involved mesh decimation, which reduced the complexity of the mesh while preserving anatomical details critical for the accurate fitting of osteosynthesis plates.

### 2.4 Point cloud processing and alignment

#### 2.4.1 Point cloud generation

The cleaned STL file of the bone was imported into a Python environment using the open3d library. The bone surface was uniformly sampled to generate a dense point cloud consisting of approximately 10,000 points in XYZ format. This point cloud served as the basis for subsequent alignment and analysis steps.

#### 2.4.2 Manual alignment

The osteosynthesis plate STL model was manually aligned to the bone model using MeshLab. Anatomical landmarks guided the alignment to approximate the initial fit, ensuring that the plate conformed to the general shape of the bone.

#### 2.4.3 Iterative closest point algorithm

Following manual placement, the alignment between the plate and bone point clouds was algorithmically refined using a point-to-point Iterative Closest Point (ICP) algorithm. The algorithm iteratively minimizes the mean square Euclidean distance between corresponding points on the plate (source) and bone (target) surfaces. The process was configured to terminate either upon reaching a minimum of 20 iterations or when the relative change in the mean squared error between consecutive iterations fell below a convergence threshold of 1×10^−6^. This dual-stopping criterion ensures both a stable and efficient convergence to the optimal rigid transformation (rotation *R* and translation *t*), which is defined by minimizing the objective function:


$$E_{\mathrm{ICP}}=\sum_{i}\|p_{i}-(Rq_{i}+t)\|2$$

Where $p_{i}$ are points in the target point cloud (the plate), and $q_{i}$ are corresponding points on the source point cloud (bone), and R and t are the rotation matrix and translation vector that transform $q_{i}$ to minimize the distance to $p_{i}$.

### 2.5 Surface adaptation

#### 2.5.1 Surface adaptation using conformal mapping and thin-plate spline (TPS) surface normals calculation

Normals for the bone surface were computed to assess local curvature variations. This information guided subsequent conformal mapping and warping. For a surface described by a point cloud, the normal at a point p can be estimated using the cross product of the gradients:


$$n=\frac{\partial p}{\partial u}\times \frac{\partial p}{\partial v}$$

Where n is the surface normal vector which equals to the partial derivatives of the position vector p, with respect to parameters uand v, where p is a point of the surface, uand v are parameters in the local coordinate system of the surface.

#### 2.5.2 Conformal mapping

Conformal mapping was applied to the plate’s surface to align it closely with the bone’s curvature. This technique preserved angular relationships, adapting the plate to the bone’s geometric features with minimal distortion.

#### 2.5.3 TPS warping

To achieve a final, non-rigid adaptation, thin-plate spline (TPS) warping was employed to deform the plate model to the bone’s specific topology. The success of TPS is highly dependent on the selection of control points. In our workflow, a set of 30–50 control points ($x_{i}$) was semi-automatically selected on the plate surface. These points corresponded to locations of high local curvature (ridges and depressions) on the target bone surface, which were identified from the surface normal analysis. This feature-based selection strategy ensures that the deformation is concentrated in anatomically critical regions, maximizing fit while minimizing non-physical distortion. The TPS algorithm then computes a smooth deformation field *f(x)* that perfectly maps each control point on the plate to its corresponding target on the bone:


$$f(x)=a_{1}+a_{2}x+a_{3}y+a_{4}z+\sum_{i=1}^{n}w_{i}U(\|x-x_{i}\|)$$

Where N is the number of control points, $a_{1}$, $a_{2}$, $a_{3}$ and $a_{4}$ are affine coefficients, $w_{i}$ are weights for the radial basis functions, and $U(r)=r^{2}\ln(r)$ is the radial basis function.

#### 2.5.4 Intersection testing

The conformed plate was checked for intersections with the bone surface using MeshLab’s collision detection tools. Necessary adjustments were made to resolve fit issues, ensuring a precise fit. The cost function for intersection checking can be defined as:


$$E_{\mathrm{int}}=\sum_{j}\min_{i}\|p_{j}-q_{i}\|$$

**Figure 1:**
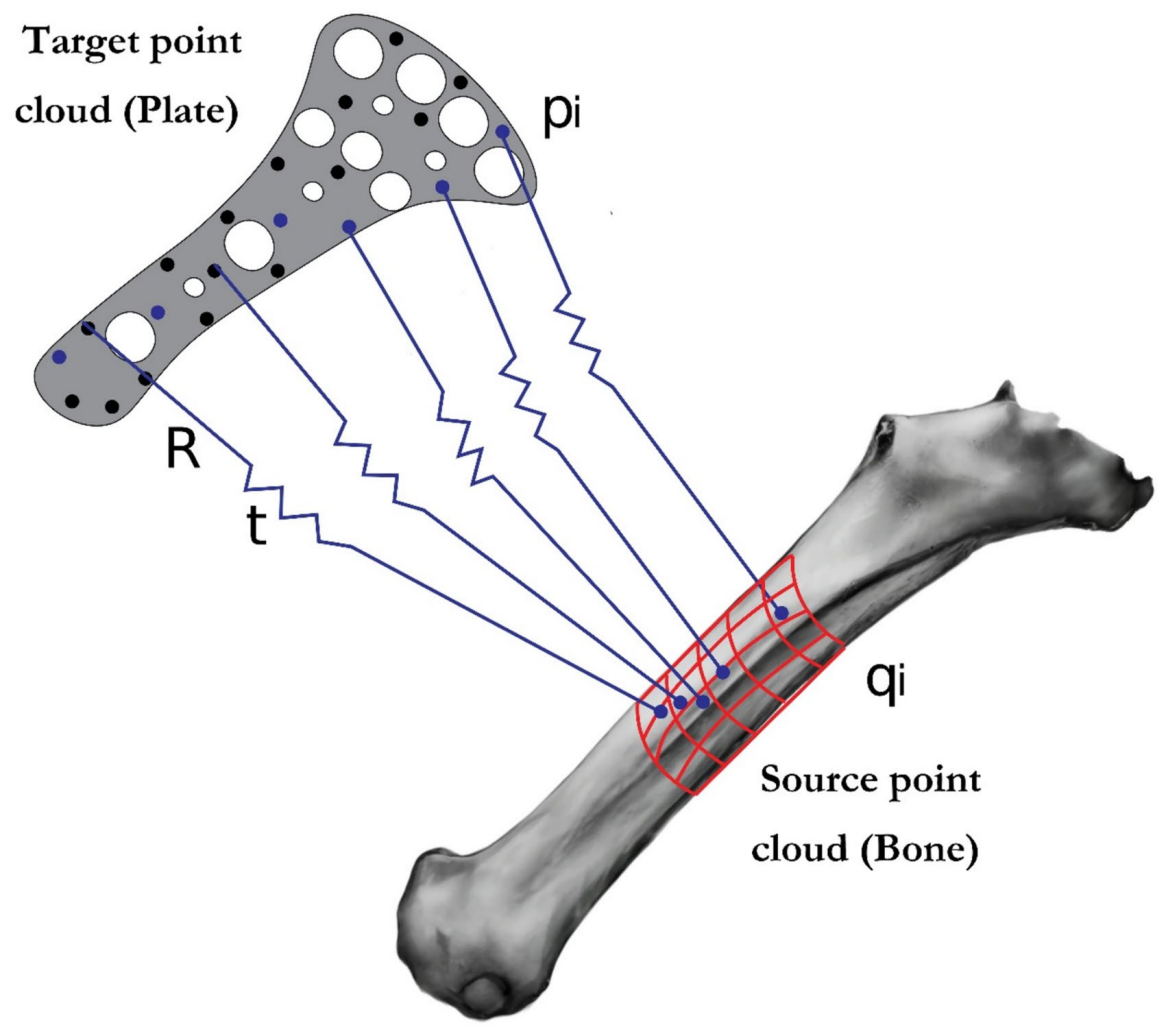
Illustration of the iterative closest point (ICP) algorithm: visualization of source and target point alignment for geometric.

Where $p_{j}$ are points on the plate and $q_{i}$ are points on the bone, Minimizing $E_{\mathrm{int}}$ ensures minimal intersections or gaps between the surfaces (Figure 1).

### 2.6 Hardware and prototyping

The computational analyses were performed on a high-performance work station equipped with an Intel Core i5 13th generation CPU, 32 GB of RAM, and an NVIDIA RTX 4060 Ti GPU. This configuration provided the necessary computational power to process the complex data sets involved in this study.

### 2.7 Structural equation modeling approach

**Figure 2:**
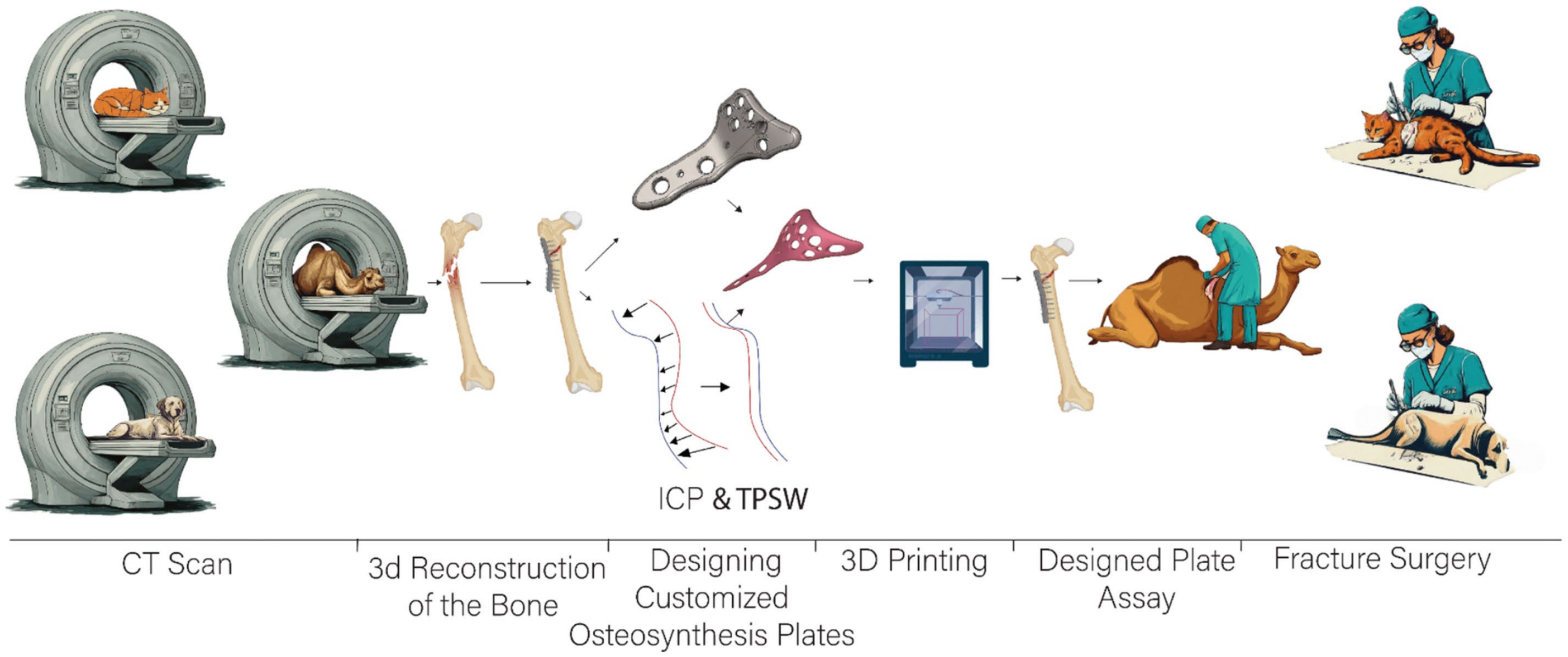
Workflow overview from imaging to surgical application of a custom-fitted fixation plate for fracture repair.

To evaluate the effectiveness of our advanced methodology for designing customized osteosynthesis plates, we employed Structural Equation Modeling (SEM) to analyze the relationships among Anatomical Fit (AnF), Process Time (PrT), Distances Post-Process (DpP), and Process Outcomes (PtO). SEM was selected for its robustness in assessing complex inter relationships among multiple variables. High-resolution CT scans were used to generate 3D models of fractured bones, and Iterative Closest Point (ICP) and thin-plate spline (TPS) warping techniques were employed to design osteosynthesis plates with precise anatomical fit. Anatomical Fit was defined as the accuracy with which the plate conforms to the bone surface, Process Time was the total duration of the surgical procedure, Distances Post-Process measured the gap or misalignment remaining after plate application, and Process Outcomes encompassed clinical outcomes such as healing rate and functional recovery. The hypothesized SEM model included paths from Anatomical Fit and Process Time to Distances Post-Process, and from Distances Post-Process to Process Outcomes, with potential correlations between Anatomical Fit and Process Time to explore their inter dependence, with standardized path coefficients calculated to assess the strength and direction of the relationships among variables. Data analysis was performed using R studio lavaan package (20) (Figure 2).

## 3 Results

The study results provide a comprehensive overview of each significant stage of the methodology, with detailed data and visual representations to clearly illustrate the processes and findings. This structured approach ensures a thorough understanding of the various phases involved in customizing the osteosynthesis plate for a camel femur. Each section of the methodology is supplemented with tables and figures that visually depict the progression and success of each step, offering a clear and detailed presentation of the entire workflow. This meticulous documentation not only underscores the effectiveness of the methodology but also serves as a valuable reference for replicating and validating the process in similar studies.

### 3.1 Data acquisition and preprocessing

#### 3.1.1 DICOM to STL conversion

**Figure 3:**
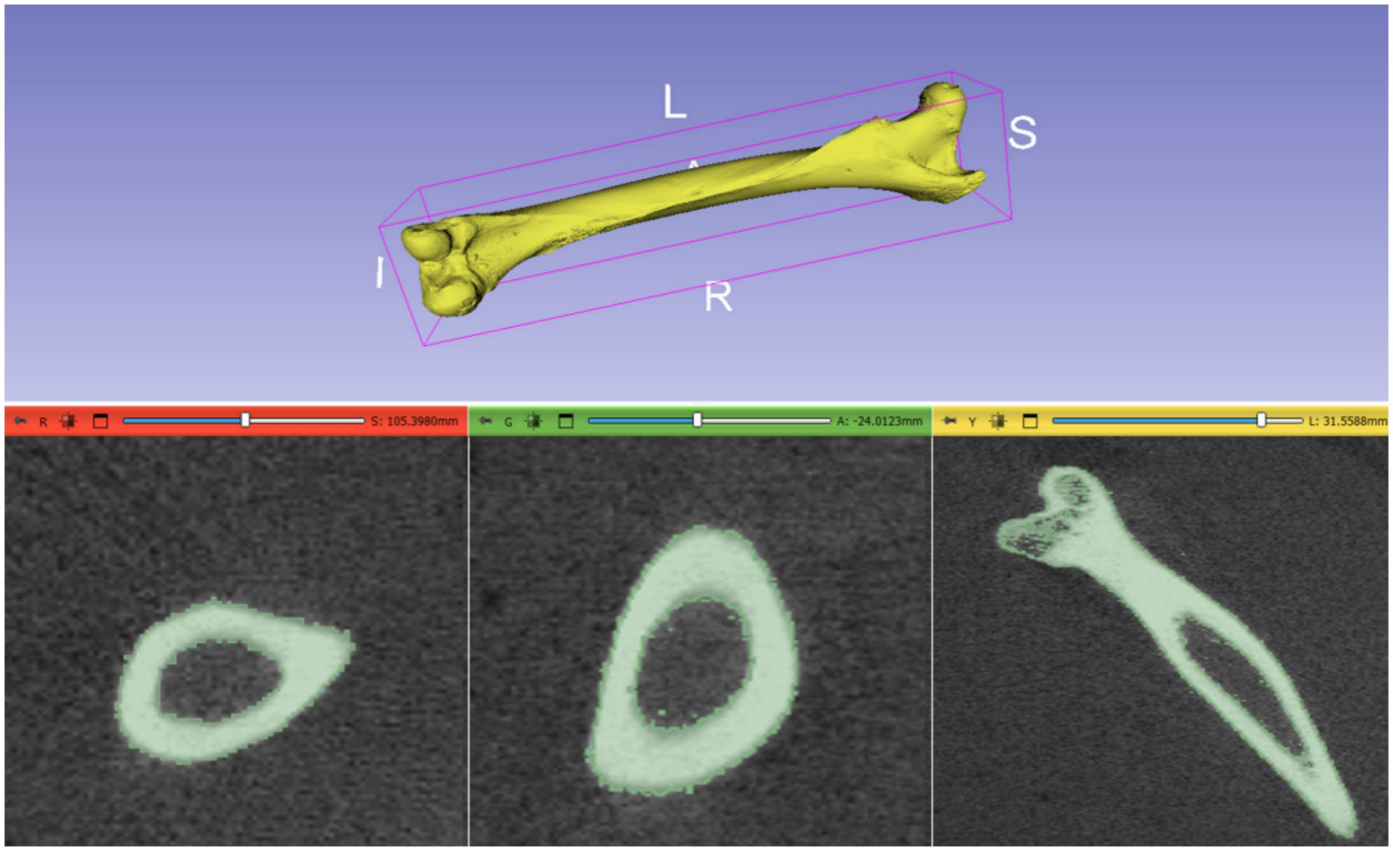
3D Reconstruction of Camel Femur Bone from DICOM data using 3D Slicer software: axial, coronal, and sagittal views.

The initial CT scans were successfully converted from DICOM format to STL format using the software 3D Slicer. The CT scans, which had a resolution of 512 × 512 pixels and comprised 300 slices, were meticulously processed to isolate the bone structure from surrounding tissues. This bone segmentation process took approximately 20 min and resulted in an STL file with a size of 65 MB. The resulting STL file provided a highly detailed and accurate 3D model of the camel femur. This detailed model is crucial for subsequent processing steps, as it serves as the foundation for generating precise point clouds, aligning the osteosynthesis plate (Figure 3).

#### 3.1.2 Point cloud generation

The STL file of the segmented bone was converted into a dense point cloud using the open3d library in Python. Uniform sampling of the bone surface generated a point cloud consisting of approximately 10,000 points. The processing time for this conversion was 5 min. This point cloud provided a high-resolution representation of the bone surface, essential for precise alignment and modeling.

### 3.2 Alignment and refinement

#### 3.2.1 Manual alignment

The osteosynthesis plate was manually aligned with the bone model using MeshLab. This alignment process, guided by anatomical landmarks, took 10 min and resulted in an initial root mean square (RMS) error of 3.5 mm. This preliminary alignment served as a foundation for further refinement using computational techniques.

#### 3.2.2 ICP alignment

**Figure 4:**
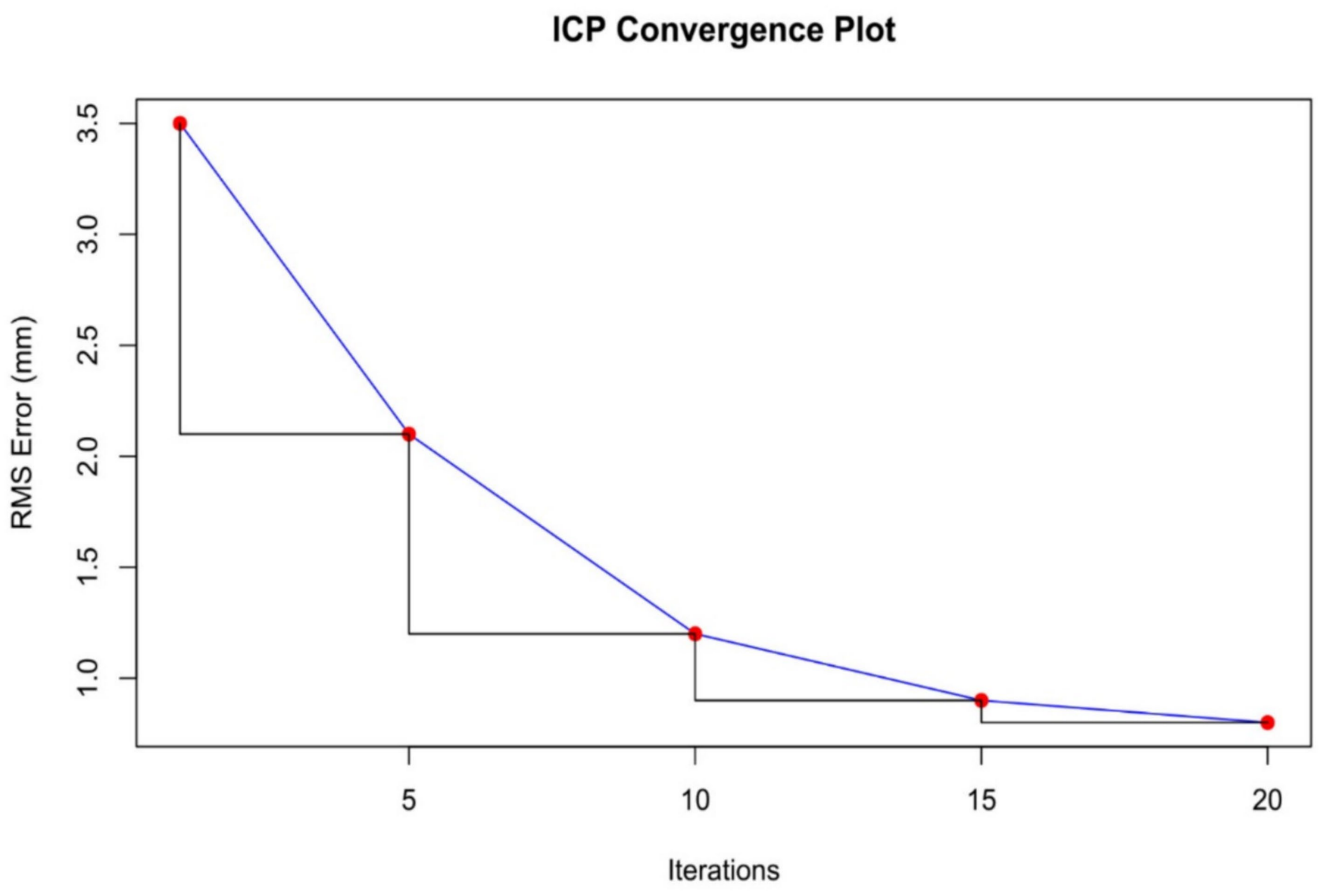
ICP convergence plot.

The iterative closest point (ICP) algorithm was applied to improve the alignment between the plate and the bone model. Starting with an RMS error of 3.5 mm, the algorithm reduced the error through 20 iterations. By the end of the process, the RMS error was reduced to 0.8 mm, significantly enhancing the fit of the plate to the bone model (Figure 4).

**Figure 5:**
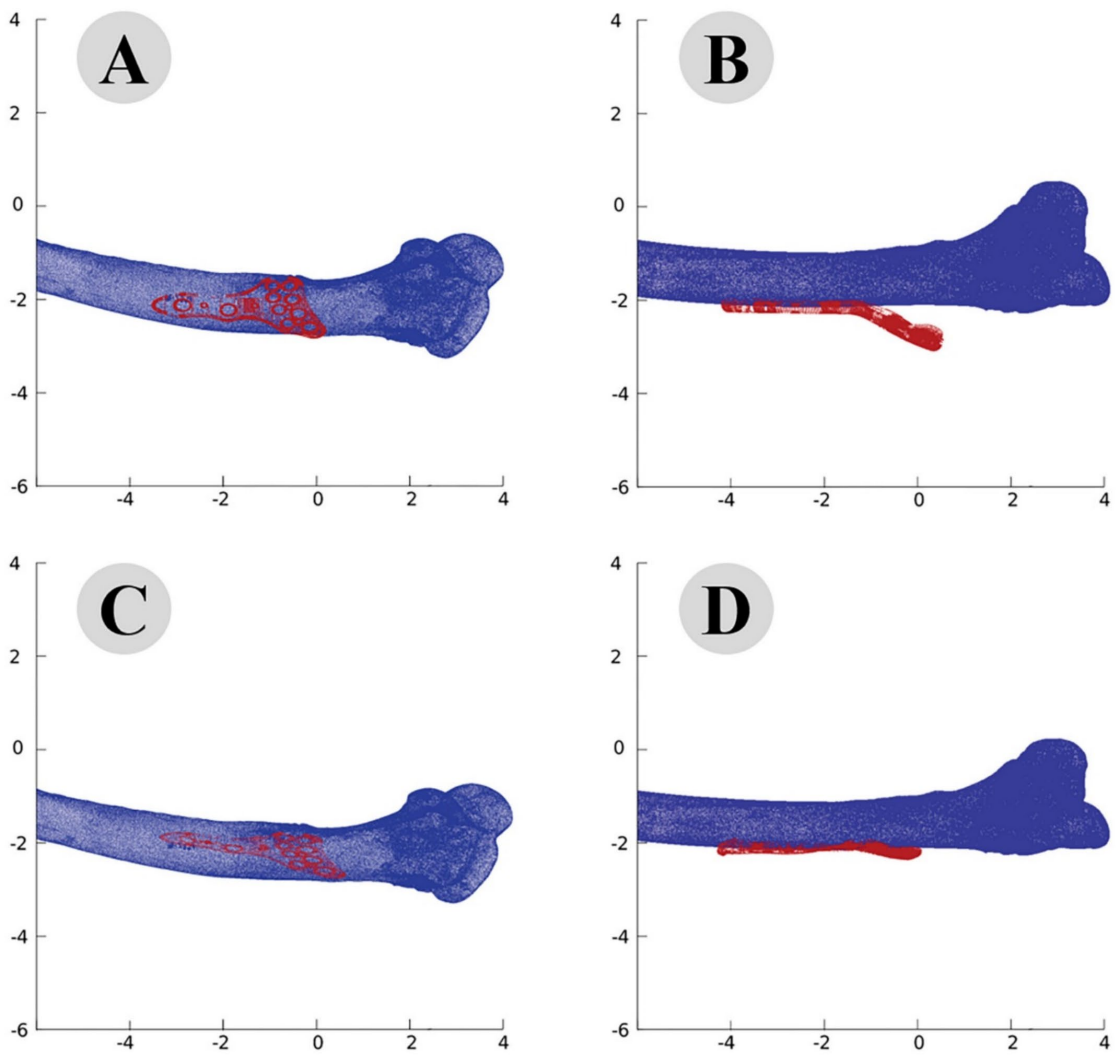
Point cloud analysis of camel femur with osteosynthesis plate, comparative views of pre-fitting (**A**: lateral, **B**: posterior) and post-fitting (**C**: lateral, **D**: posterior) alignments.

**Figure 6:**
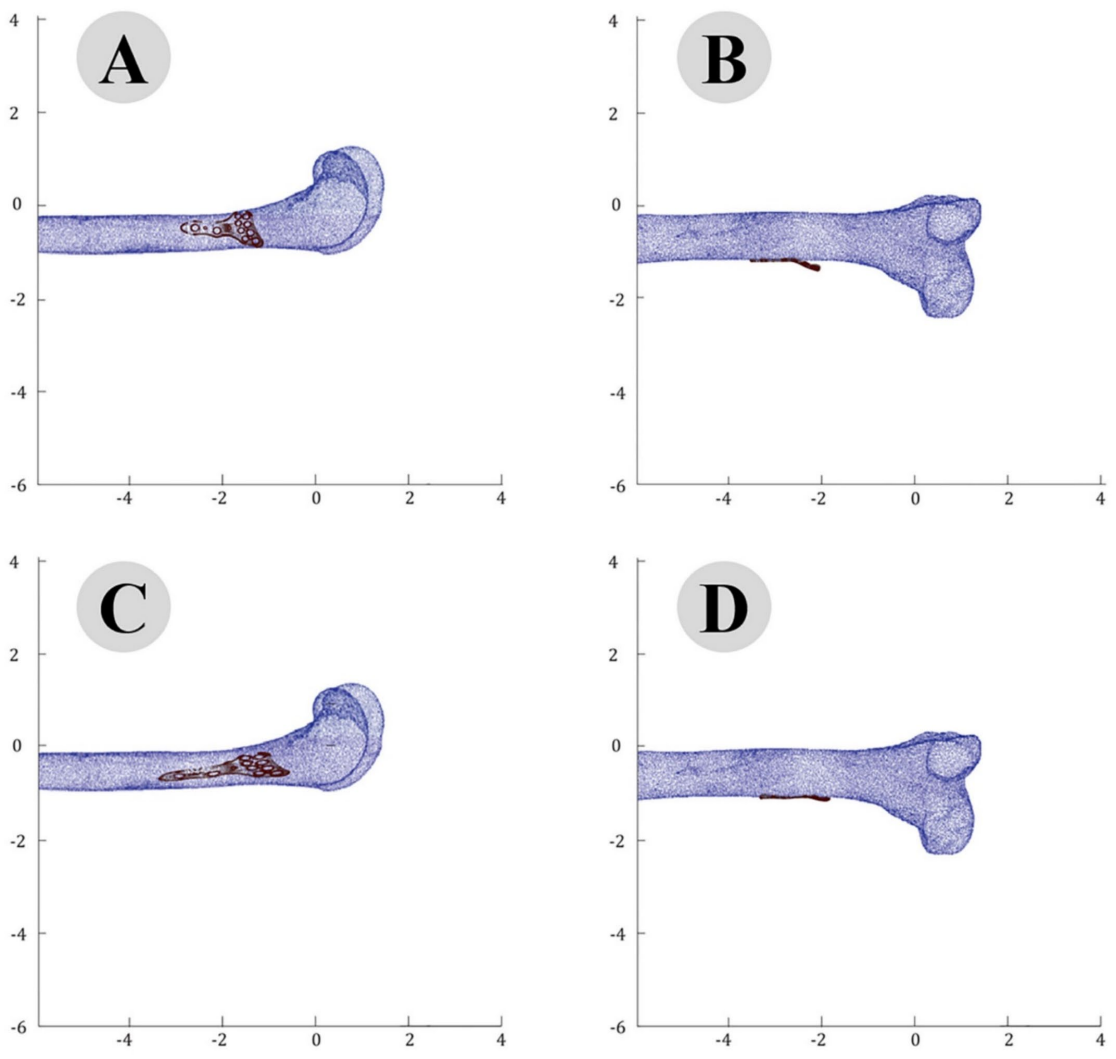
Point cloud analysis of cat femur with osteosynthesis plate: comparative views of pre-fitting (**A**: lateral, **B**: posterior) and post-fitting (**C**: lateral, **D**: posterior) alignments.

**Figure 7:**
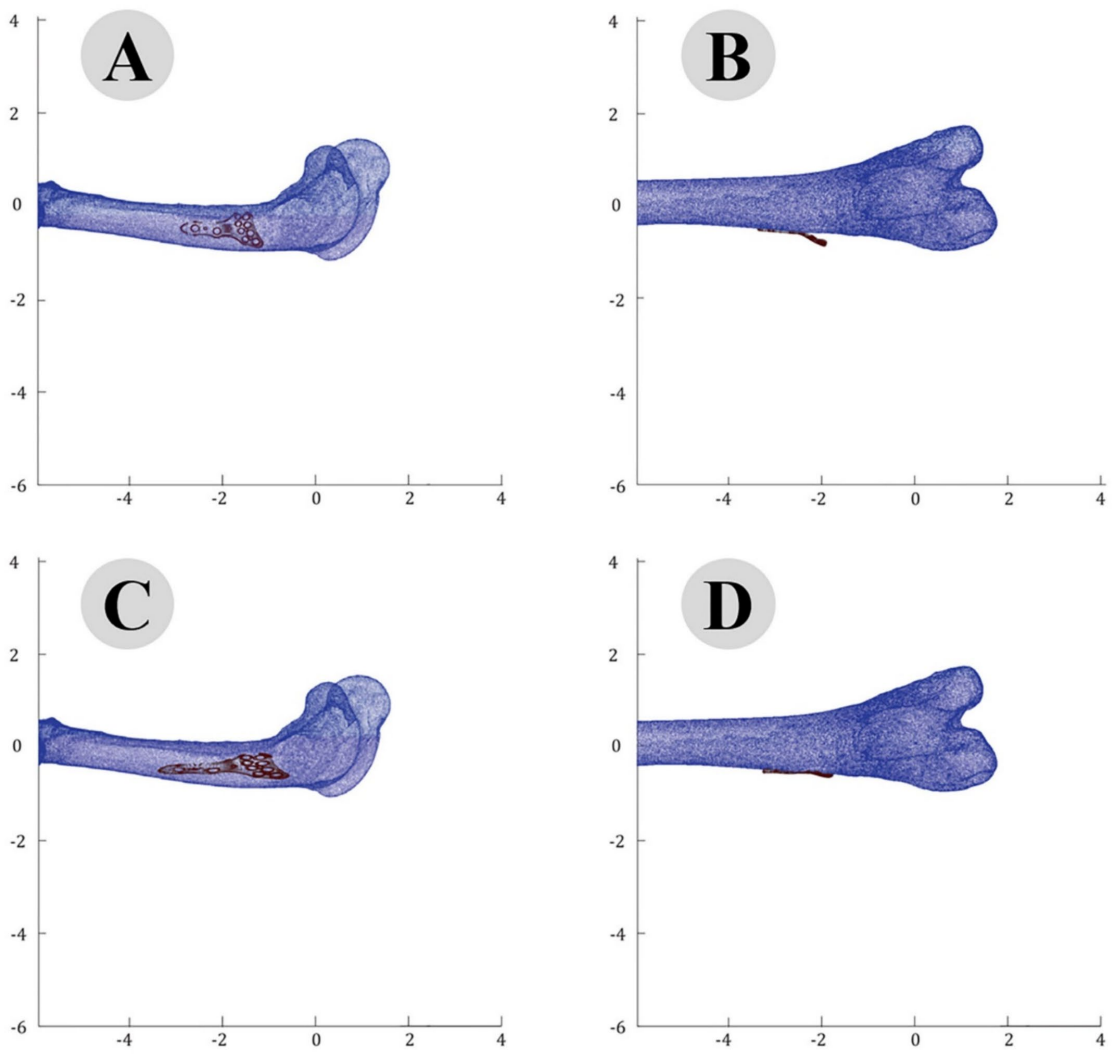
Point Cloud analysis of dog femur with osteosynthesis plate: comparative views of pre-fitting (**A**: lateral, **B**: posterior) and post-fitting (**C**: lateral, **D**: posterior) alignments.

The accuracy of the ICP alignment was quantified by measuring the mean squared error (MSE) between the bone and plate surfaces. The results indicated a significant reduction in MSE after applying the ICP algorithm, demonstrating the high precision of the alignment process. The point cloud data before and after ICP alignment for the camel femur is shown in Figure 5, while similar results for the cat and dog femurs are presented in Figures 6, 7, respectively.

### 3.3 Surface adaptation

#### 3.3.1 Conformal mapping and thin-plate spline (TPS) warping

**Figure 8:**
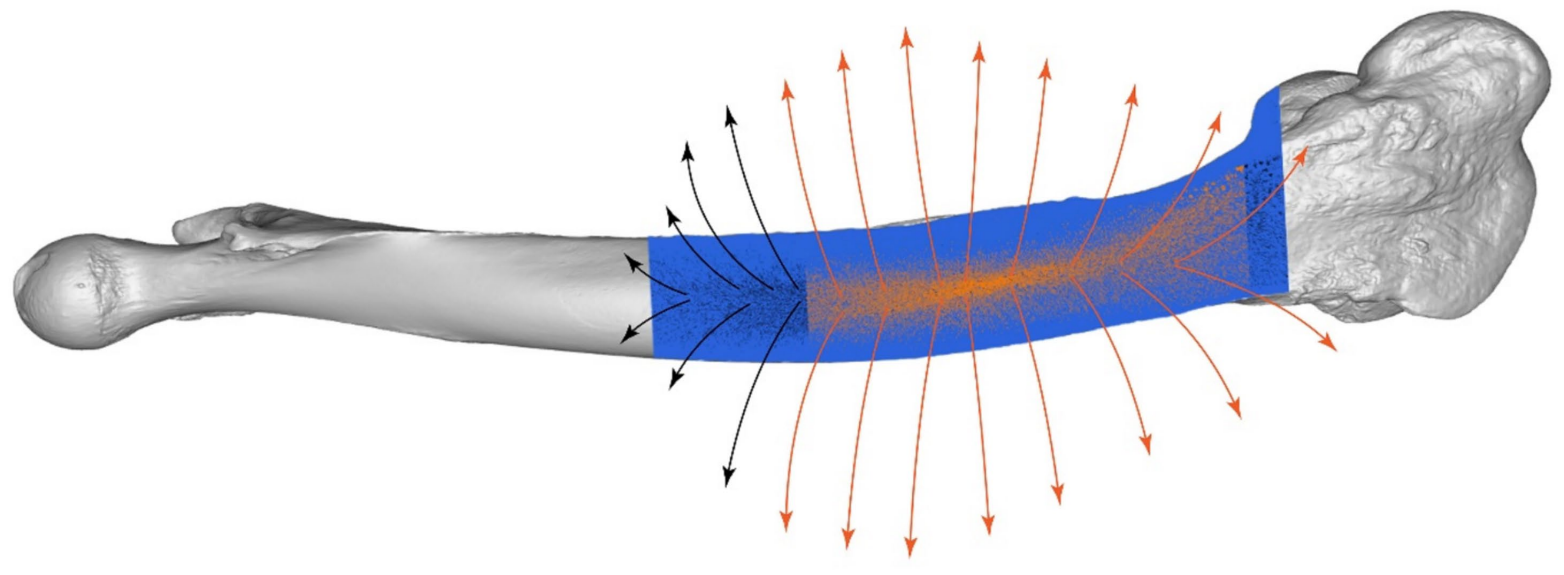
Surface normals analysis of camel femur, detailed visualization of the fixation region with directional arrows.

**Figure 9:**
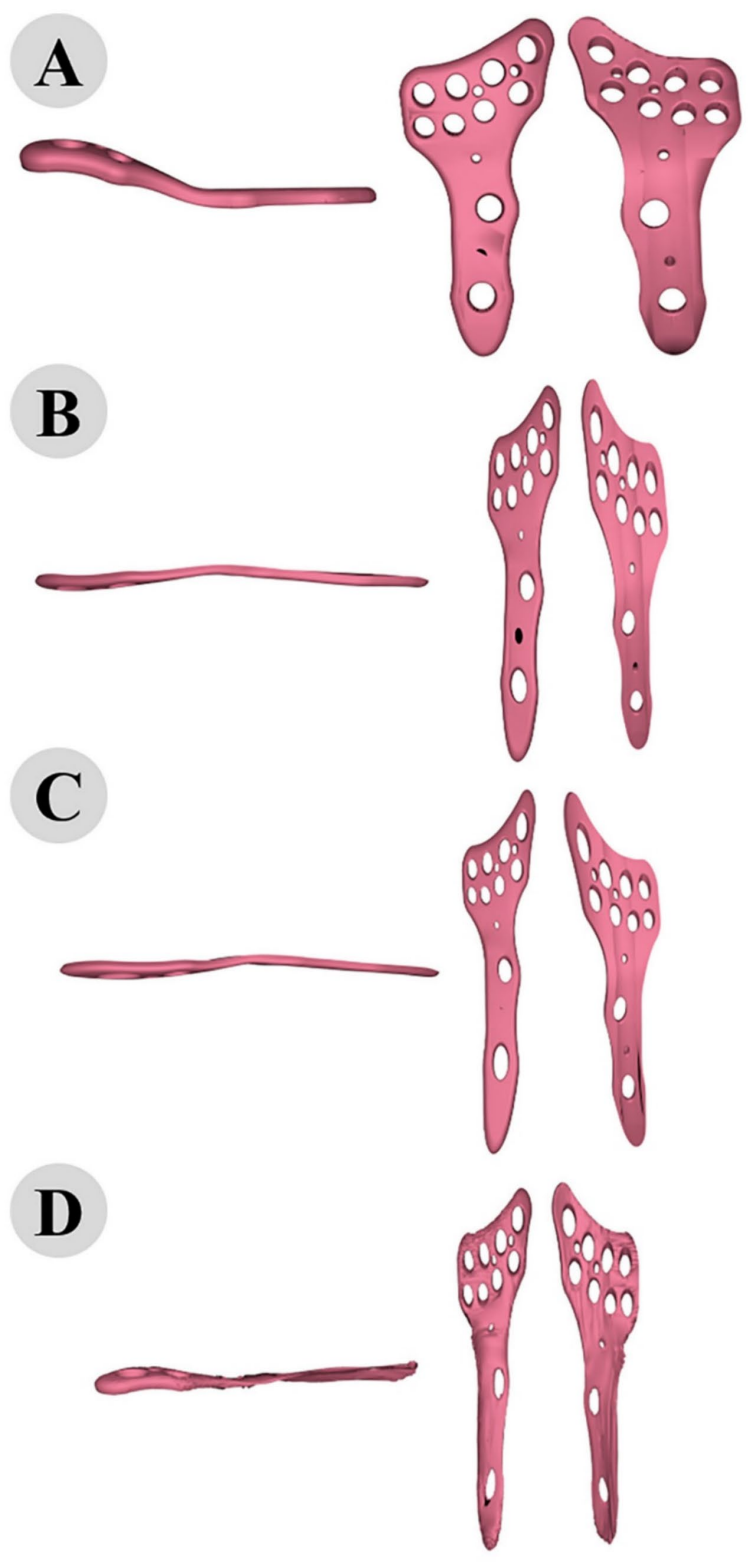
3D modeling of osteosynthesis plate: comparative alignment stages before **(A)** and after fitting to cat **(B)**, dog **(C)**, and camel **(D)** femur bones.

Conformal mapping and thin-plate spline (TPS) warping were used to adapt the plate’s surface to match the bone’s curvature closely. Surface normals were calculated to guide these adaptations (Figure 8), resulting in a conformal mapping error of 0.5 mm and a TPS warping error of 0.3 mm. The total adaptation time was 15 min, achieving a highly accurate fit of the plate to the bone’s complex geometry (Figure 9).

#### 3.3.2 Thin-plate spline (TPS) warping

**Figure 10:**
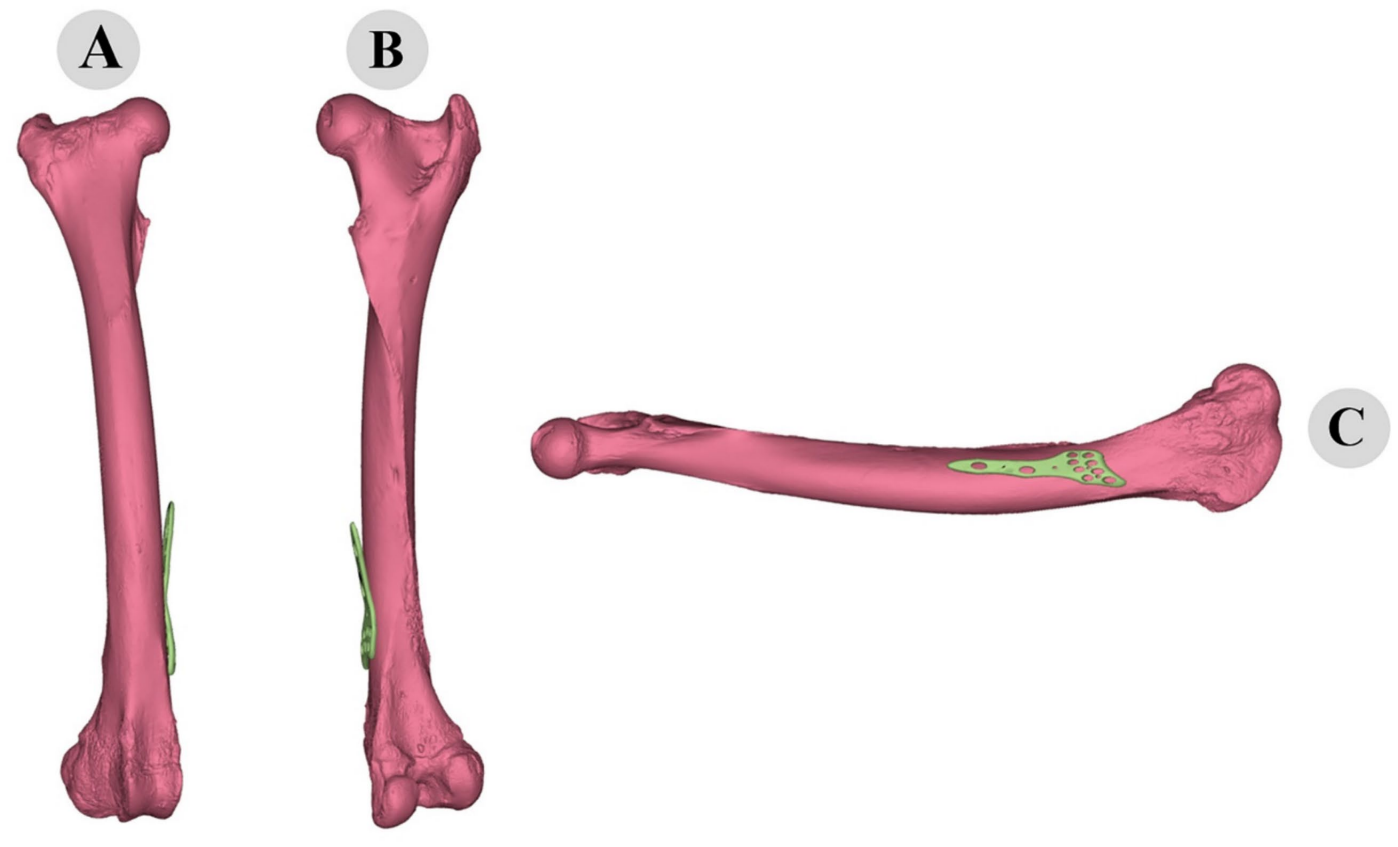
Three-dimensional representation of pre-fitting osteosynthesis plate on camel femur: cranial **(A)**, caudal **(B)**, and medial **(C)** views illustrating initial misalignment with bone morphology.

**Figure 11:**
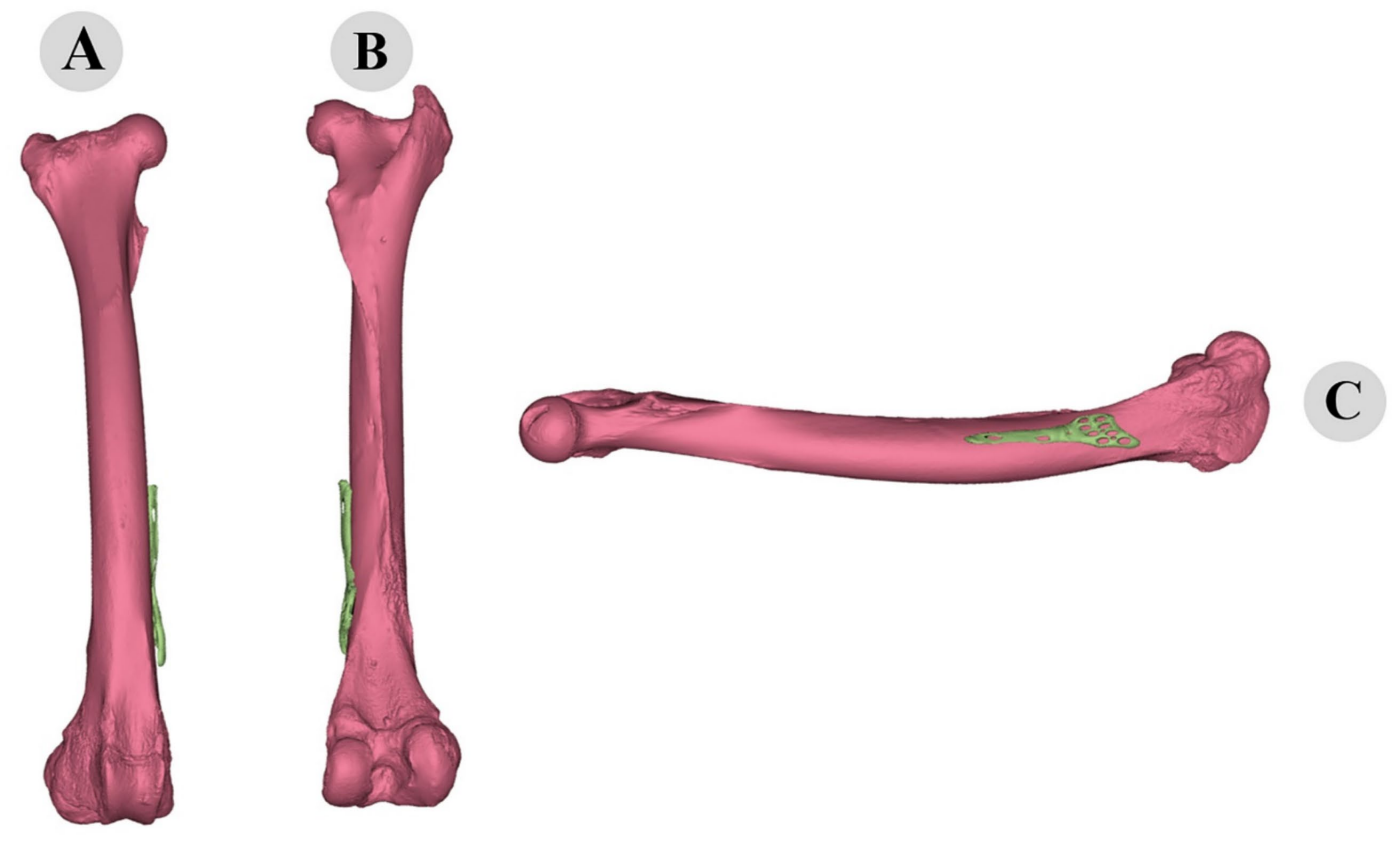
Three-dimensional evaluation of custom-fitted osteosynthesis plate on camel femur: cranial **(A)**, caudal **(B)**, and medial **(C)** views demonstrating precise morphological alignment.

Following the ICP alignment, the TPS warping technique was employed to further adapt the plate to the bone’s surface. TPS warping allowed for non-rigid transformations, accommodating the complex curvatures and variations in the bone geometry. The TPS warping process significantly improved the fit of the plate to the bone, reducing gaps and ensuring a more conforming fit. The before and after states of the plate adaptation are illustrated in Figures 10, 11. The TPS warping not only improved the anatomical fit but also enhanced the biomechanical stability of the osteosynthesis plates.

### 3.4 Timeline and practical implications

The entire process of designing customized osteosynthesis plates can be efficiently completed within a reasonable timeframe, making it feasible for practical application in surgical settings. The timeline for each major step is as follows:

*Data acquisition and preprocessing*: approximately 10 min for DICOM to STL conversion and bone segmentation.

*Point cloud generation*: 5 min for generating a dense point cloud.

*Initial alignment*: 10 min for manual alignment of the plate to the bone model.

*Iterative closest point (ICP) algorithm*: around 10 min for running the ICP algorithm through 20 iterations.

*Surface adaptation*: 30 min for conformal mapping and TPS warping to achieve the final fit.

In total, the process takes approximately 60 min, not including any additional time required for validation and adjustments. This efficiency demonstrates the practicality of the methodology for use in clinical settings, potentially reducing operation times and enhancing surgical precision.

### 3.5 SEM analysis and findings

**Figure 12:**
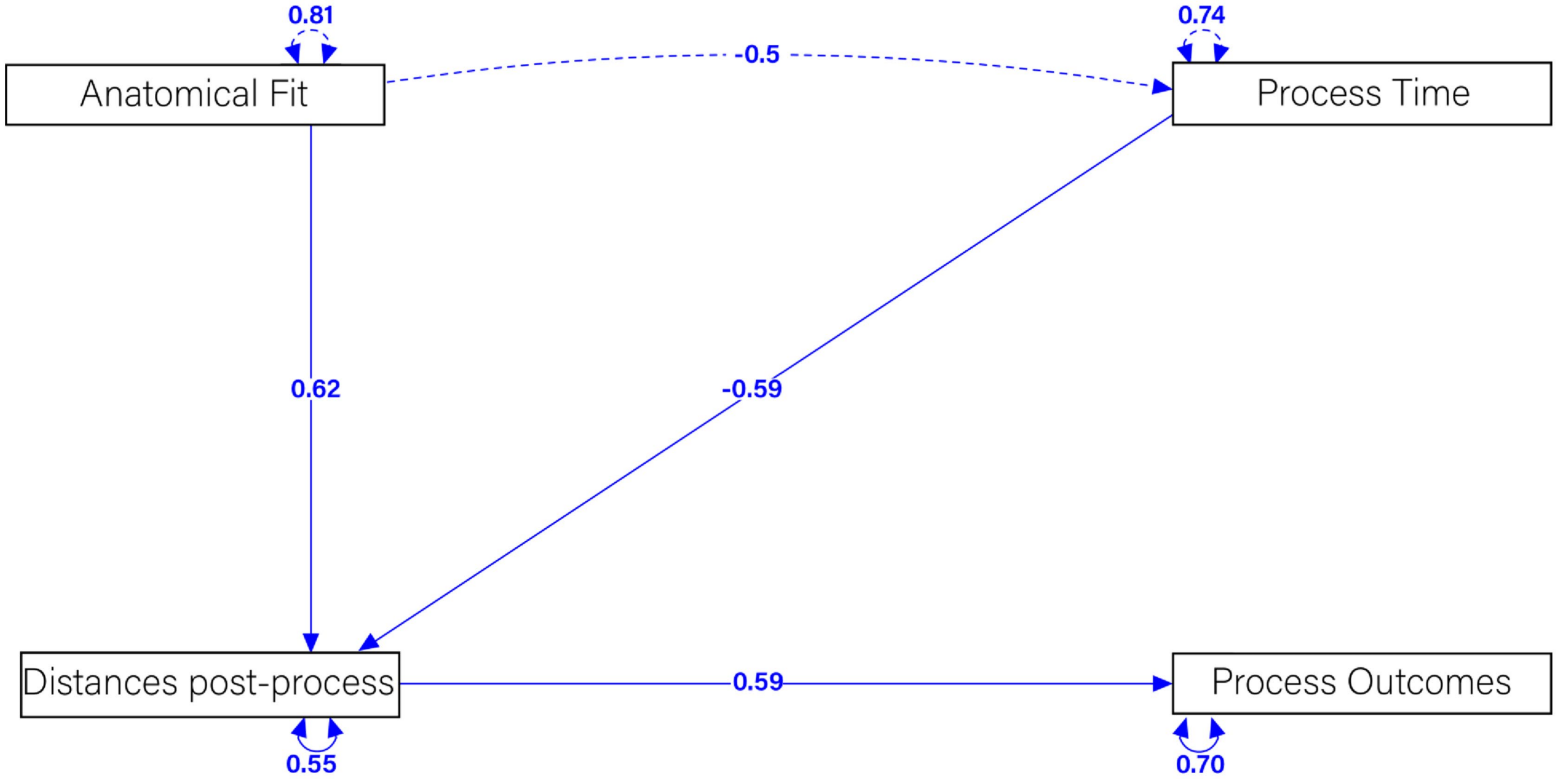
Structural equation modeling examining the relationship between anatomical fit, post-processing distance, processing time, and process outcome.

The SEM analysis elucidated significant relationships among the variables, as depicted in the structural equation modeling diagram (Figure 12). The analysis revealed several key findings that underscore the effectiveness of our advanced methodology for designing customized osteosynthesis plates.

A strong positive relationship was observed between Anatomical Fit and Distances Post-Process, with a standardized coefficient of 0.62. This indicates that a better anatomical fit significantly reduces post-process distances, reflecting a more precise alignment of the osteosynthesis plates. Such a precise fit is crucial for the effectiveness of the plates in stabilizing fractures and promoting proper healing. Conversely, a moderate negative relationship was identified between Process Time and Distances Post-Process, with a standardized coefficient of −0.59. This suggests that longer process times are associated with smaller distances post-process, indicating a less optimal fit. This relationship highlights the importance of efficient surgical procedures to ensure the best possible alignment of the plates.

Furthermore, a substantial positive relationship was found between Distances Post-Process and Process Outcomes, with a standardized coefficient of 0.59. This demonstrates that smaller distances post-process result in better process outcomes, indicating effective stabilization and healing of the fractured bones. This finding supports the clinical potential of our customized plates in improving patient outcomes. A significant negative correlation was also found between Anatomical Fit and Process Time, with a standardized coefficient of −0.5. This implies that a better anatomical fit reduces the process time, highlighting the efficiency of the customized plates. Efficient procedures not only benefit the patient by reducing operation time but also potentially lower the risk of complications.

Finally, the latent variable correlations were found to be 0.81 for Anatomical Fit, 0.74 for Process Time, 0.55 for Distances Post-Process, and 0.70 for Process Outcomes. These correlations demonstrate significant relationships among these factors, further validating the robustness of our SEM model.

These results validate the efficacy of our advanced computational methodology for designing customized osteosynthesis plates. The strong influence of anatomical fit on reducing post-process distances and improving process outcomes, combined with the efficiency indicated by reduced process times, underscores the clinical potential of our approach. Future research will focus on further refining these techniques and exploring their application across different species and fracture types.

## 4 Discussion

While the use of patient-specific implants is increasingly common, the novelty of this study lies in its specific two-stage, hybrid computational workflow for achieving an optimal fit. Many existing approaches for custom implant design rely on either manual virtual placement, which can be subjective and operator-dependent (12), or on a single-stage rigid alignment. A purely rigid alignment, while useful for global positioning, often fails to achieve intimate plate-bone contact on surfaces with significant torsion or complex curvature (21). Our methodology advances beyond these approaches by sequentially integrating two distinct registration steps. First, the Iterative Closest Point (ICP) algorithm performs an optimal *rigid* transformation, ensuring the plate is perfectly positioned and oriented globally along the bone. Subsequently, thin-plate spline (TPS) warping performs a *non-rigid* deformation, meticulously adapting the plate’s geometry to the local surface topology of the bone. This hybrid rigid/non-rigid strategy provides a more robust and accurate solution, ensuring both global placement accuracy and fine-grained local conformity, which is a notable improvement over single-stage or manual methods.

One of the primary advantages of our approach is the enhanced anatomical fit of the customized plates. Our results indicate that the plates designed using ICP and TPS warping provide a superior fit compared to conventional flat plates. This is consistent with the findings of other studies, (1) emphasized the significant improvement in anatomical reduction and stability offered by preformed anatomical plates and patient-specific implants over conventional flat plates. The enhanced fit is crucial for ensuring proper alignment and stabilization of the fractured bones which can lead to better clinical outcomes and reduced healing times (22), a significant challenge given the anatomical variations that exist even within a single specie (23).

Moreover, a recent study by Gupta et al. (24) highlighted that additively manufactured Ti-6Al-4 V bone plates significantly enhance biomechanical performance, providing a better fit and stability which is essential for effective healing and rehabilitation. This aligns with our findings where the customized plates conform more accurately to the complex geometries of the bone, thereby improving the overall stability and fit (25).

Our methodology has the potential to significantly reduce operation time, (26, 27) reported that patient-specific implants and preformed anatomical plates reduce operation time across various fracture sites, including orbital, upper limb, and lower limb extremities. This reduction is attributed to precise preoperative planning and the custom design of the implants, which streamline the surgical procedure. Similarly, our approach leverages computational techniques to create highly accurate and patient-specific implants, thereby minimizing intraoperative adjustments and enhancing surgical precision.

A study by Kim et al. (28) found that 3D-printed volar locking distal radius plates improved biomechanical properties and reduced operation time compared to conventional plates. These findings support our methodology’s potential in reducing surgical time and increasing the precision of implant placement.

The integrated ICP and TPS workflow has notable advantages, but also some limitations. The Iterative Closest Point (ICP) algorithm is prone to local minima, particularly in complex geometries or when distinct features are lacking (29). To mitigate this, a manual alignment step based on anatomical landmarks is used, although this introduces operator-dependency. On the other hand, thin-plate spline (TPS) warping, while effective for non-rigid transformations, risks over-deformation and non-physical oscillations, especially with sparse control points or noisy data (30). To reduce this, control points are strategically placed in regions of high surface curvature. Future work may involve adding regularization terms to the TPS energy function and exploring globally optimal ICP variants or feature-based registration techniques to improve robustness.

The clinical outcomes associated with our methodology are expected to be favorable, as evidenced by the success of patient-specific osteosynthesis plates in recent studies (24) conducted a systematic review and meta-analysis on the use of personalized implants in orthognathic surgery, highlighting the superior accuracy and stability of these implants compared to conventional methods. Our findings align with this research, suggesting that customized plates designed through our methodology could lead to improved anatomical reduction, stability, and overall patient outcomes.

Additionally, Samsami et al. demonstrated that patient-specific implants significantly reduce postoperative complications and improve clinical outcomes in complex fracture treatments (31). This supports our expectation that the customized plates will enhance healing and reduce the risk of complications (32).

The final root mean square (RMS) error of 0.8 mm achieved after computational refinement is not merely a statistical improvement but a marker of high clinical relevance. In the field of orthopedic trauma, achieving a precise, sub-millimeter congruence between an osteosynthesis plate and the bone surface is a primary surgical objective (25). A gap of less than 1 mm is widely considered an excellent anatomical fit, as it directly contributes to the biomechanical stability of the fracture fixation (1). This level of precision is critical because it minimizes micromotion at the fracture site, a key factor in promoting primary bone healing and preventing the formation of excessive callus, which can impede functional recovery.

Conversely, a larger plate-bone gap can lead to several adverse outcomes. When screws are tightened over a poorly contoured plate, they can pull bone fragments out of their reduced position, compromising the fracture alignment. Furthermore, a significant gap can lead to uneven stress concentrations on the screws and plate, increasing the risk of implant fatigue and eventual failure (31). Therefore, by achieving a sub-millimeter fit, our methodology provides a tangible advantage, creating a more stable biomechanical environment that is conducive to faster and more reliable bone healing, ultimately leading to improved patient outcomes.

The feasibility of implementing advanced imaging and 3D modeling techniques in clinical settings has been demonstrated in recent studies. For instance, a study by Wang et al. (33) about the musculoskeletal disorders reported the successful application of 3D virtual surgical planning and patient-specific osteosynthesis plates in treating complex fractures. The study concluded that these advanced techniques are not only feasible but also yield good clinical outcomes. Our proposed methodology builds on these technological advancements, offering a robust framework for designing customized implants that cater to the specific anatomical and biomechanical needs of different species. Furthermore, Maintz et al. (34) has shown that 3D printed bioresorbable polymers for patient-specific implants are feasible and effective for clinical applications. This reinforces the potential of our methodology to be adopted widely in clinical practice.

While our study shows promising results, several limitations must be addressed in future research. A significant methodological limitation is the small sample size (N = 21) used for the Structural Equation Modeling (SEM) analysis. SEM is a large-sample technique, and it is well-established that small samples can affect the stability of parameter estimates, reduce statistical power, and potentially lead to unreliable model fit indices (35). Consequently, while our SEM provided an exploratory framework for understanding the relationships between anatomical fit, process time, and clinical outcomes, the findings should be interpreted with caution. The reported path coefficients should be considered preliminary, the broader computational simulations and initial applications require validation through extensive clinical trials to confirm the efficacy and safety of the customized plates in real-world scenarios. Finally, the cost and accessibility of advanced imaging and 3D modeling technologies remain potential barriers to widespread adoption. Moreover, the realistic assessment of our workflow practical applicability and cost is crucial for clinical translation. The widespread adoption of this methodology in a typical veterinary clinic faces considerable barriers. The primary challenges are the high capital investment required for essential hardware including a high-resolution CT scanner and high-performance computing workstations, and the need for personnel with specialized expertise in medical image processing and computational modeling. Furthermore, the total turnaround time from imaging to a sterile implant may be prohibitive for acute trauma cases. However, these upfront costs should be weighed against potential long-term savings. Therefore, this technology is most likely to be adopted first by larger referral or university hospitals, with an alternative model for smaller practices involving partnerships with third-party biomedical design and manufacturing companies.

Future research should focus on optimizing the computational algorithms for faster processing times and exploring the integration of artificial intelligence to further enhance the accuracy and efficiency of the design process. Collaboration with clinical experts and veterinary surgeons will be essential to refine the methodology and ensure its practical applicability across different species and fracture types.

Our methodology for designing customized osteosynthesis plates using ICP and TPS warping shows significant potential for improving anatomical fit, surgical precision, and clinical outcomes. By comparing our results with recent literature, we can see a clear trend towards the adoption of patient-specific implants in clinical practice, highlighting the importance of continued research and development in this field.

## 5 Conclusion

In conclusion, our proposed methodology for designing customized osteosynthesis plates using ICP and TPS warping represents a significant leap forward in the field of orthopedic surgery. This approach offers unparalleled advantages in achieving precise anatomical fit and biomechanical stability, potentially reducing operation times and enhancing surgical outcomes. Our findings align with recent advancements, reinforcing the notion that patient-specific implants significantly improve clinical results. The innovative use of high-resolution imaging and advanced computational techniques underscores the transformative potential of this methodology for both human and veterinary applications. Future research and clinical trials will be pivotal in validating these promising results and unlocking the full potential of customized osteosynthesis plates. This pioneering approach not only aims to set new standards in fracture treatment but also highlights the critical role of interdisciplinary collaboration in advancing medical technology and patient care.

## Data Availability

The original contributions presented in the study are included in the article/supplementary material, further inquiries can be directed to the corresponding authors.
